# Aggressive intraoperative warming and postoperative pulmonary complications in elderly patients recovering from esophageal cancer surgery: sub-analysis of a randomized trial

**DOI:** 10.3389/fmed.2023.1157392

**Published:** 2023-07-14

**Authors:** Xiaofei Lu, Qiliang Jiang, Yuwei Qiu, Wei Tang, Daniel I. Sessler, Jingxiang Wu

**Affiliations:** ^1^Department of Anesthesiology, Shanghai Chest Hospital, Shanghai Jiao Tong University, School of Medicine, Shanghai, China; ^2^Outcomes Research Consortium, Cleveland, OH, United States; ^3^Department of Outcomes Research, Anesthesiology Institute, Cleveland, OH, United States

**Keywords:** anesthesia, surgery, elderly patients, body temperature management, postoperative pulmonary complications

## Abstract

**Background:**

Elderly patients having esophagectomies often become hypothermic which may promote complications. We tested the hypothesis that aggressive warming to a core temperature of 37°C reduces postoperative pulmonary complications (PPCs) in elderly patients having esophageal cancer resections.

**Methods:**

This study was a pre-defined sub-study of a multi-center, parallel group, superiority trial (PROTECT). Patients aged >65 years and having elective radical resection of esophageal cancer in a single center were randomly allocated into either aggressive warming group (target intraoperative core temperatures of 37°C) or routine thermal management group (target intraoperative core temperatures of 35.5°C). The primary endpoint was the incidence of PPCs. Secondary endpoints included duration of chest tube drainage and other postoperative complications.

**Results:**

A total of 300 patients were included in the primary analysis. PPCs occurred in 27 (18%) of 150 patients in the aggressive warming group and 31 (21%) of 150 patients in the routine thermal management group. The relative risk (RR) of aggressive versus routine thermal management was 0.9 (95% CI: 0.5, 1.4; *p* = 0.56). The duration of chest drainage in patients assigned to aggressive warming was shorter than that assigned to routine thermal management: 4 (3, 5) days vs. 5 (4, 7) days; hazard ratio (HR) 1.4 [95% CI: 1.1, 1.7]; *p* = 0.001. Fewer aggressively warmed patients needed chest drainage for more than 5 days: 30/150 (20%) vs. 51/150 (34%); RR:0.6 (95% CI: 0.4, 0.9; *p* = 0.03). The incidence of other postoperative complications were similar between the two groups.

**Conclusion:**

Aggressive warming does not reduce the incidence of PPCs in elderly patients receiving esophagectomy. The duration of chest drainage was reduced by aggressive warming. But as a secondary analysis of a planned sub-group study, these results should be considered exploratory.

**Clinical trial registration:**

https://www.chictr.org.cn/showproj.aspx?proj=37099, ChiCTR1900022257.

## 1. Introduction

Esophageal cancer is the eighth most common cancer and the sixth leading cause of cancer-related death worldwide (1). Surgical resection remains the mainstay of treatment. However, esophagectomy is among the most invasive gastrointestinal operations, with high postoperative morbidity and mortality even with less invasive approaches (2). Postoperative pulmonary complications (PPCs) remain the most common postoperative morbidity in these patients (3), with a reported incidence of 26–48% in elderly patients (4–7).

Various risk factors are associated with PPCs including advanced age, anemia, hypoalbuminemia, and intraoperative bleeding (8). Elderly patients are especially susceptible to PPCs due to coexistence of chronic respiratory disorders, reduced lung reserve, small airway collapse, along with other anatomical and physiological changes that accompany aging. Hypothermia may be an additional risk factor.

Intraoperative hypothermia, conventionally defined as a core body temperature below 36°C, is common in unwarmed surgical patients with an incidence ranged from 50 to 90% (9). Patients having esophagectomies are especially likely to become hypothermic because much of the body is exposed and various position changes are often required (10). Moderate intraoperative hypothermia (e.g., 34.5°C) causes clinical complications including surgical site infection, bleeding, shivering and delayed acute recovery (11–14).

Unwarmed patients having major surgery often have final intraoperative core temperatures of 35.5°C or less. Whether aggressive warming to maintain the core temperature near 37°C reduces pulmonary complications in elderly patients having esophagectomies remains unknown. We therefore tested the primary hypothesis that aggressive warming to a core temperature of 37°C reduces in-hospital PPCs in elderly patients. Secondarily, we tested the hypotheses that aggressive warming improves gas exchange, reduces extra-pulmonary complications, reduces chest tube drainage, speeds chest tube removal, and shortens ICU stays and the duration of hospitalization.

## 2. Methods

This study was a pre-defined sub-study of the PROTECT trial (15). PROTECT was a multicenter, parallel group, superiority trial that randomized 5,056 patients from 12 sites in China and at the Cleveland Clinic in United States to target intraoperative core temperatures of 35.5°C or 37°C. The primary outcome was myocardial injury and cardiovascular complications; secondary outcomes were surgical site infection and transfusion.

Our sub-study was restricted to patients at the Shanghai Chest Hospital who were aged >65 years and had elective radical resection of esophageal cancer expected to last at least 2 h. The PROTECT study and this sub-study were approved by the Ethics Committee of Shanghai Chest Hospital (Institutional Review Board #KS1905), and all patients provided written consent. The trial was registered before patient enrollment at chictr.org.cn (ChiCTR1900022257, primary investigator: Jingxiang Wu, date of registration: April 1, 2019, https://www.chictr.org.cn/showproj.aspx?proj=37099). This manuscript adheres to the applicable CONSORT guidelines.

We enrolled patients who were scheduled for elective esophagectomy lasting at least 2 h, planned overnight admission, and had available skin surface for warming >50%. All patients had total minimally invasive esophagectomy (MIE) performed with neck or intrathoracic anastomosis via one of the three surgical approaches: laparoscopic trans-hiatal, laparoscopic-thoracoscopic McKeown type 3-incision, or laparoscopic-thoracoscopic Ivor-Lewis approach. We excluded patients who had a body mass index (BMI) exceeding 30 kg/m^2^ or who required dialysis.

### 2.1. Randomization and core temperature management

Patients were randomly assigned through a computer-generated randomization sequence at a 1:1 ratio with random-sized blocking, to either aggressive or routine thermal management. To conceal allocation, investigators accessed a web-based site about 1 h before surgery. The exposure in this study refers to the difference in thermal management strategies between the two groups. It was not possible to mask patients with prewarming and clinicians with intraoperative warming. Patients and clinicians were thus aware of group assignment. Postoperative measurements were made by an independent team of researchers who were not informed of the patients’ assignment.

Temperature of operating rooms were maintained at 21°C ± 1°C and humidity at 40 to 60%. All intravenous fluids (including transfused fluid and blood) were warmed to 42°C via a fluid warmer (Smiths Medical ASD Inc., Rockland, MA) in both groups. Nasopharyngeal temperature was monitored continuously during the operation with the probe inserted at least 10 cm (16).

Patients assigned to aggressive warming group were pre-warmed with a full-body forced-air cover for about 30 min before anesthetic induction. During surgery, the patients were aggressively heated with two forced-air covers to a target core temperature of 37°C. In the routine thermal management group, patients were not pre-warmed. Warming was limited to transfused fluid or blood. Forced-air warming was only used if nasopharyngeal temperature decreased to <35.5°C. The warming systems were Bair Hugger 750 (Arizant Healthcare Inc., Eden Prairie, MN, United States) and Cocoon Convective Warming CWS 4000 (Care Essentials Ltd., North Geelong, Australia).

### 2.2. Anesthesia methods and monitoring

All patients were monitored with 5-lead electrocardiography (ECG), noninvasive blood pressure (NIBP), pulse oximetry (SpO_2_), and partial pressure of end-tidal CO_2_ (PetCO_2_), invasive arterial blood pressure, and central venous pressure (GE Healthcare Finland Oy, Helsinki, Finland). Total intravenous anesthesia with muscle relaxation was used in all patients. After preoxygenation, anesthesia was induced with target-controlled infusion of propofol (target plasma concentration 3.0–4.0 μg/mL). Sufentanil (0.5–1.0 μg/kg) and cisatracurium (0.2 mg/kg) were injected to facilitate the tracheal intubation.

After tracheal intubation, the patients were ventilated with a tidal volume of 6 mL/kg, respiratory rate of 12–14 min^−1^, inspiration/expiration ratio of 1:2, and fractional of inspired oxygen tension (FiO_2_) of 50–100%. End-tidal carbon dioxide pressure (PetCO_2_) was maintained 35–45 mm Hg during most of surgery, but was allowed to increase to 42–55 mm Hg during carbon dioxide pneumothorax.

Anesthesia was maintained with 2–4 μg/mL propofol and 2–4 ng/mL remifentanil with target-controlled infusion, along with cisatracurium infused at 0.12 mg·kg^−1^·hour^−1^. The infusion rates were titrated to maintain normal vital signs. Intraoperative and postoperative arterial blood gasses were measured as necessary.

A consistent analgesic regimen was used for all enrolled patients in this study. Multimodal analgesia was adopted in patients undergoing thoracoscopic esophageal cancer resection, including ultrasound-guided thoracic paravertebral block combined with postoperative patient-controlled intravenous analgesia (PCIA). 0.5% ropivacaine was used for T4-T6 paravertebral block, and the intravenous analgesia formula of PCIA was sufentanil 0.8-1 μg/kg and dezocine 0.3 mg/kg (mixed with 0.9% normal saline to a total of 100 mL analgesic solution). The PCIA was programmed to deliver a 0.5 mL bolus on demand, with a lock-out interval of 15 min, and a background infusion rate of 2 mL/h.

The primary outcome was the incidence of PPCs during hospitalization. The Melbourne Group Scale version 2 (MGS-2) was used to screen for the presence of PPCs. MGS-2 is a diagnostic scoring tool for PPCs based on chest X-ray, white cell count, fever, purulent sputum, microbiology, oxygen saturations, physician diagnosis, and intensive therapy unit (ITU)/high-dependency unit readmission (17). A PPC was diagnosed when at least 4 of the 8 factors were present.

The secondary outcomes included (1) incidence of extrapulmonary complications following surgery including neurological (perioperative stroke, postoperative cognitive dysfunction, postoperative delirium and epilepsy), cardiovascular (arrhythmia, myocardial ischemia, myocardial infarction, heart failure, cardiac arrest), hematological complications, and severe hepatic and renal dysfunction (2); volumes of chest drainage at 1, 2, 3, and 5 days after surgery, the time to remove chest tubes, and prolonged drainage defined as chest tube drainage lasting longer than 5 days. The decision on when to remove chest tubes was made by the surgical team, who were blinded to the division of the groups (3); duration of stay in the intensive care unit (ICU); and (4) the length of hospital stay after surgery.

### 2.3. Statistical analysis

SPSS 25.0 (IBM, United States) and R version 4.1.3 was used for data analysis. Continuous variables were tested for normal distribution, and data showing a normal distribution are expressed as the means ± SDs. Data showing a skewed distribution were expressed as medians [interquartile range]. Categorical variables were expressed as *n* (%).

We used absolute standardized differences to assess baseline characteristics and were calculated using JASP 0.8.6 software (University of Amsterdam, the Netherlands). Per Austin we considered factors having standardized differences exceeding $1.96\times \sqrt{\frac{1}{n1}+\frac{1}{n2}}$ = 0.23 as imbalanced (18). Differences in the incidence of PPCs and postoperative extrapulmonary complications between groups were assessed using the *χ*^2^ or Fisher exact tests. Relative risk (RR) and 95% CI were calculated. Secondary outcomes, including the time to chest drainage removal, postoperative length of stay and ICU stay, were compared with Mann–Whitney U tests.

Repeated-measures analysis of variance with post-hoc Bonferroni correction was used to evaluate changes in body temperature and chest drainage volume across time within groups. Kaplan–Meier analysis was used to evaluate the time to chest tube removal, with the log-rank test used to test for differences. *p* < 0.05 was considered statistically significant.

### 2.4. Sample size estimate

Previous studies report that 26–48% of elderly patients experience PPCs after esophagectomy (4–7). Our pilot study suggested that up to 30% (3/10) of patients receiving curative resection of esophageal carcinoma experienced postoperative PPCs with routine thermal management while the incidence of PPCs was 20% (2/10) with aggressive intraoperative warming. We assumed that aggressive warming management could reduce PPCs from 30 to 20%, thus a sample size of 290 patients had 80% power to detect a 5% two-sided significance using R version 4.1.3. Considering anticipated dropouts and variation in group differences, we set the sample size at 300 patients.

## 3. Results

**Figure 1:**
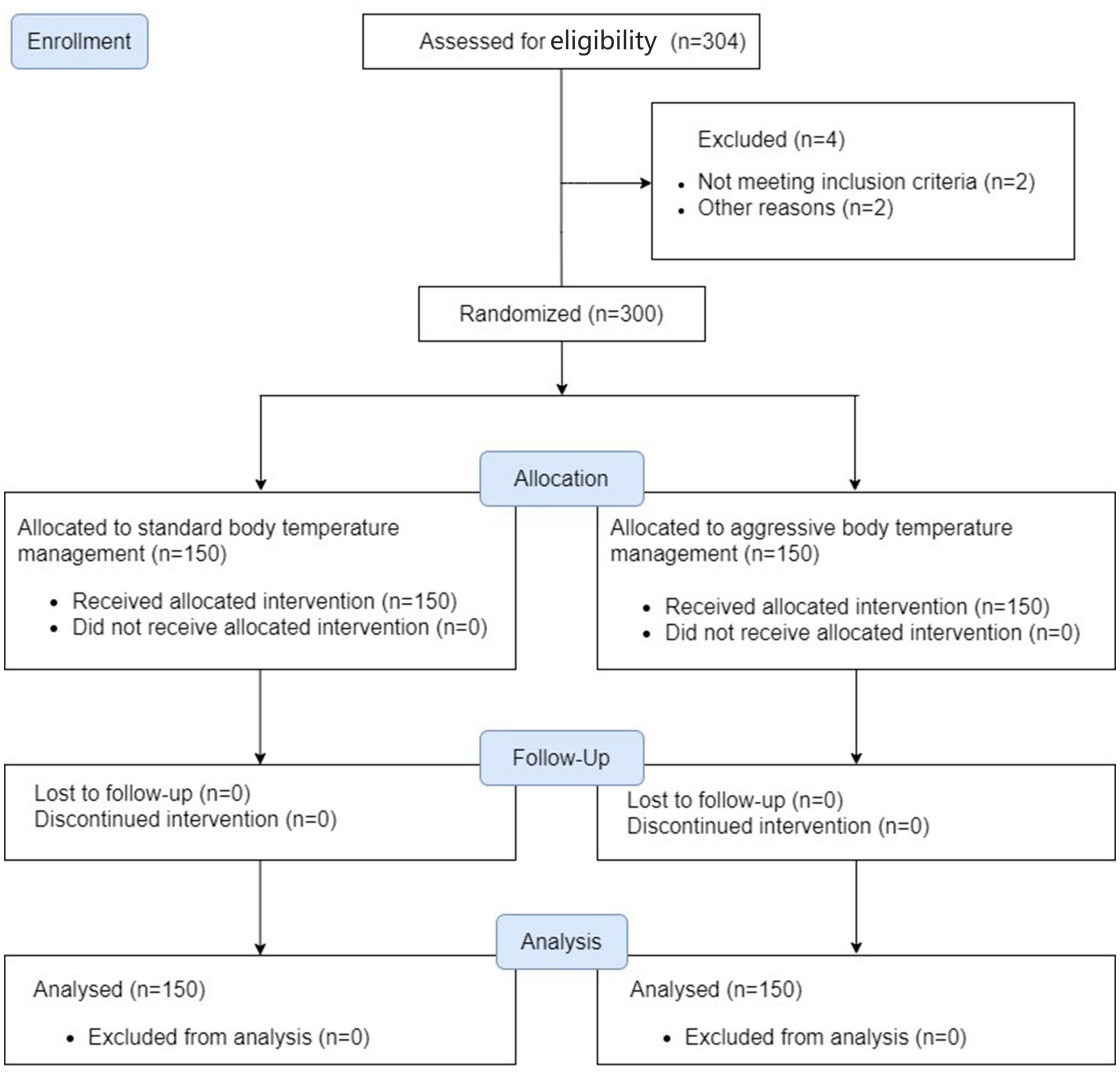
Enrollment and analysis flow chart.

We enrolled 150 patients per group between August 2019 and February 2021 when the underlying PROTECT trial finished. All patients completed the trial and were included in the analysis (Figure 1). Baseline characteristics were balanced (Table 1).

**Table 1:** Pre–and intraoperative patient characteristics.

Characteristics	Aggressive intraoperative warming (*n* = 150)	Routine thermal management (*n* = 150)	Absolute standardized difference
Age, years	71 ± 5	70 ± 4	0.22
Sex			
Male, *n* (%)	114 (76%)	124 (83%)	0.17
Female, *n* (%)	36 (24%)	26 (17%)	
Body mass index (kg/m^2^)	23 ± 3	22 ± 3	0.19
ASA			
II, *n* (%)	68 (45%)	81 (54%)	0.17
III, *n* (%)	82 (55%)	69 (46%)	
Smoker, *n* (%)	31 (21%)	35 (23%)	0.06
Preoperative chemotherapy, *n* (%)	27 (18%)	31 (21%)	0.07
Preoperative radiotherapy *n* (%)	18 (12%)	14 (9%)	0.09
Comorbidity, *n* (%)			
COPD	17 (11%)	21 (14%)	0.08
Diabetes mellitus	43 (29%)	30 (20%)	0.20
Hypertension	73 (49%)	64 (43%)	0.12
Arrhythmia	6 (4%)	4 (3%)	0.07
Coronary heart disease	2 (1%)	4 (3%)	0.10
Preoperative pulmonary function			
FEV1, L	2.2 ± 0.6	2.3 ± 0.6	0.19
FEV1/FVC (%)	75.8 ± 11.0	75.2 ± 11.4	0.05
MEF75, L/s	5.0 ± 2.0	5.3 ± 2.0	0.12
MEF50, L/s	2.5 ± 1.1	2.5 ± 1.0	0.05
MEF25, L/s	0.7 ± 0.4	0.7 ± 0.3	0.01
MVV, L/min	72.6 ± 22.5	74.5 ± 21.8	0.08
RV/TLC (%)	50.3 ± 8.6	49.8 ± 8.7	0.06
DLCO, mL/mmHg/min	16.5 ± 4.3	16.5 ± 4.3	0.01
Surgical duration, min	332 ± 77	320 ± 90	0.15
One-lung ventilation time, min	111 ± 49	114 ± 49	0.05
Blood loss, mL	199 ± 72	198 ± 93	0.01
Blood transfusion, mL	0 [0, 0]	0 [0, 0]	0.14
Intraoperative crystalloids, mL	1,228 ± 345	1,207 ± 379	0.05
Intraoperative colloids, mL	915 ± 294	862 ± 311	0.14

Data are presented as *n* (%), means ± SDs, or medians [interquartile range]. ASA, American Society of Anesthesiologists; FVC, forced vital capacity; FEV1, forced expiratory volume in 1 sec; MEF, maximal expiratory flow; MVV, maximum ventilatory volume; RV, residual volume; TLC, total lung capacity; DLCO, diffusion capacity for carbon monoxide of the lung. COPD, Chronic obstructive pulmonary disease.

### 3.1. Intraoperative core temperature

**Figure 2:**
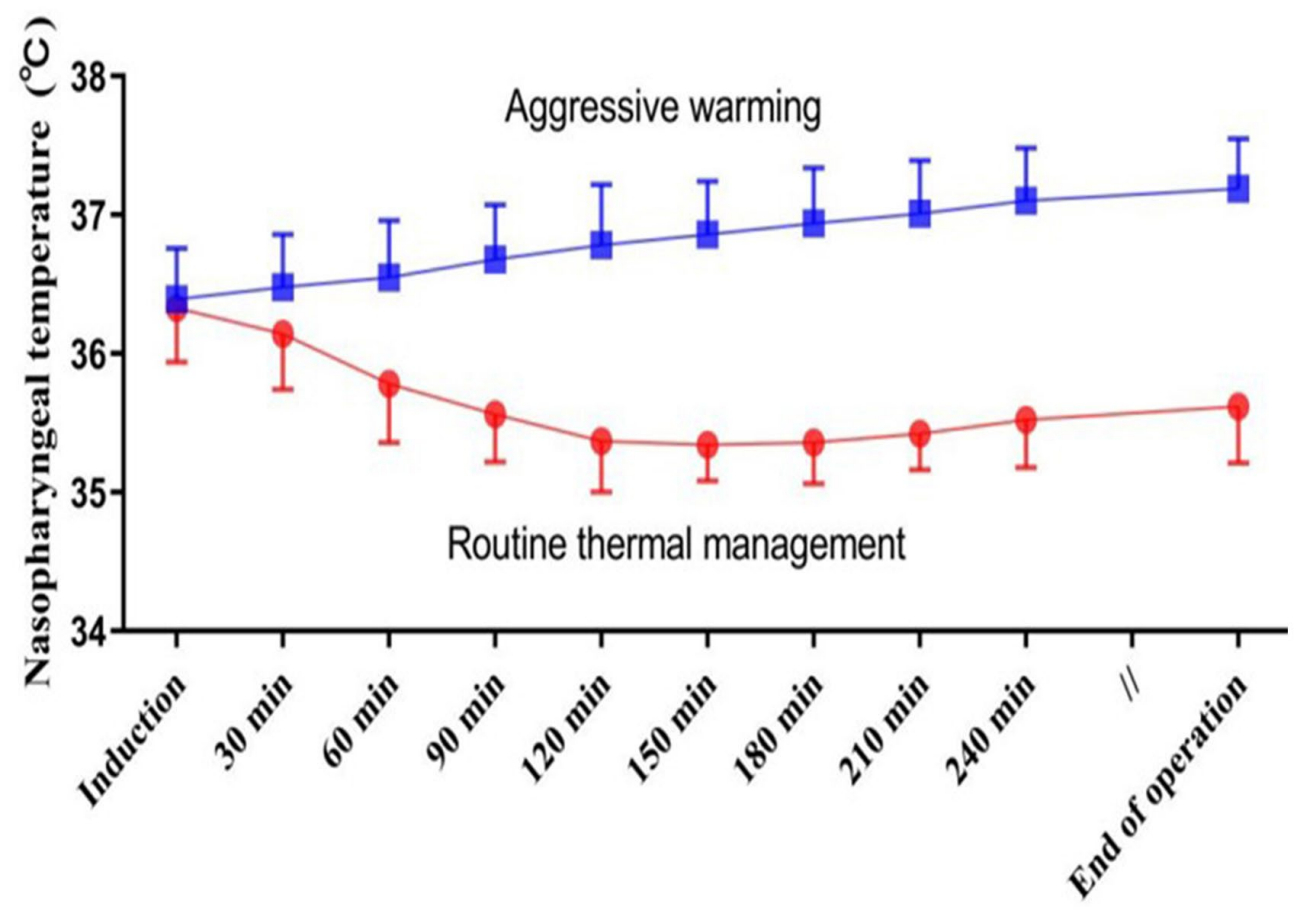
Intraoperative nasopharyngeal temperatures in each thermal management group. Results are presented as means and SDs.

Before anesthetic induction, the core temperatures were similar between the two groups. After anesthesia induction, the core temperature significantly reduced in the routine thermal group, particularly within the first 120 min, and subsequently fluctuated around 35°C (Figure 2). Target temperatures were well-maintained during the surgery which averaged 5.4 ± 1.4 h. Core temperature at the end of surgery was 35.6 ± 0.4°C in patients assigned to a target of 35.5°C, and was 37.1 ± 0.5°C in those assigned to a target temperature of 37°C (Figure 2).

### 3.2. Postoperative pulmonary complications

**Figure 3:**
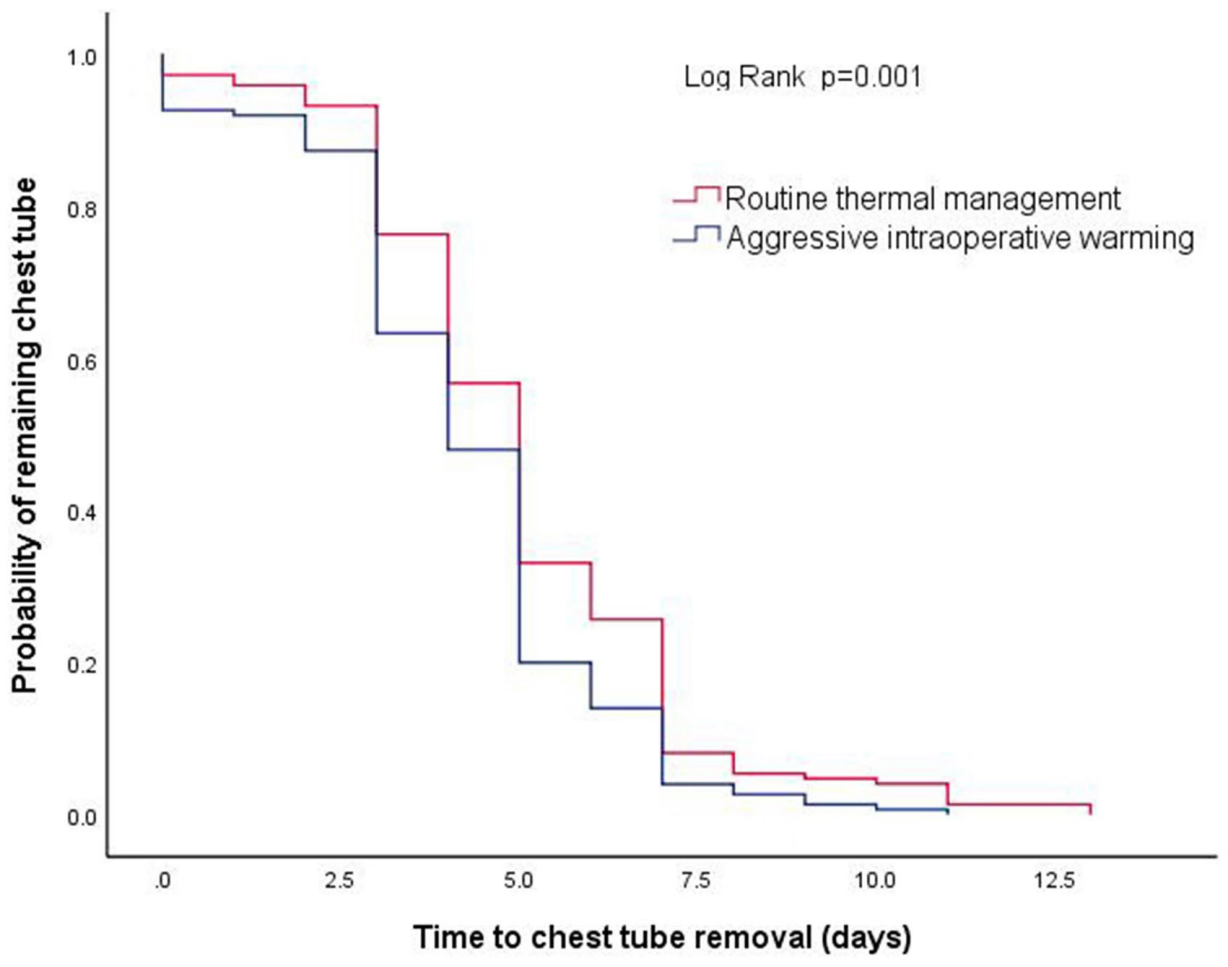
Kaplan–Meier curve for chest tube duration. Chest tube drainage time in patients assigned to routine versus aggressive thermal management. Log-rank test, *p* = 0.001.

A total of 58 of 300 (19%) patients developed PPCs. The incidence of PPCs was 18% (27/150) in patients with aggressive thermal management and 21% (31/150) in patients with routine thermal management, with the difference neither clinically meaningful nor statistically significant: RR 0.9 [95% CI: 0.5, 1.4]; *p* = 0.56 (Table 2). There were 30 patients (20%) in the aggressive warming group and 51 patients (34%) in the routine group having chest tube drainage lasting longer than 5 days: RR 0.6 [95% CI: 0.4, 0.9]; *p* = 0.03. The median chest tube drainage time was 4 (3, 5) days in the aggressive intraoperative group, compared to 5 (4, 7) days in the routine thermal management group. A Kaplan–Meier analysis with a log-rank test showed that patients receiving aggressive thermal management had a shorter chest tube drainage time: hazard ratio (HR) 1.4 [95% CI, 1.1, 1.7]; *p* = 0.001 (Figure 3). Patients assigned to aggressive warming had lower chest tube drainage volumes on postoperative days (Table 3). Based on the medical history data, we carried out a retrospective analysis of the postoperative platelet levels in the 300 patients. Over time, the postoperative platelet levels in both groups initially decreased and then increased, but there was no significant difference between the two groups (Table 4).

**Table 2:** PPCs using the Melbourne group scale (MGS-2) diagnostic criteria.

MGS-2 Criteria	Aggressive intraoperative warming (*n* = 150)	Routine thermal management (*n* = 150)	RR [95% CI]	*p*-value
PPCs	27 (18%)	31 (21%)	0.9 [0.5, 1.4]	0.56
MGS 2 score	2 [2, 3]	2 [2, 3]		0.71
Temperature > 38°C, *n* (%)	40 (27%)	40 (27%)	1.0 [0.7, 1.5]	1.00
White cell count >11.2 or use of respiratory antibiotics, *n* (%)	102 (68%)	90 (60%)	1.1 [1.0, 1.3]	0.15
Physician diagnosis of pneumonia or chest infection, *n* (%)	7 (5%)	12 (8%)	0.6 [0.2, 1.4]	0.24
Chest X-ray findings of atelectasis/consolidation, *n* (%)	9 (6%)	14 (9%)	0.6 [0.3, 1.4]	0.28
Incidence of postoperative production of purulent (yellow/green) sputum different from preoperative sputum, *n* (%)	74 (49%)	93 (62%)	0.8 [0.6, 1.0]	0.03*
Positive results upon sputum microbiological analysis, *n* (%)	10 (7%)	11 (7%)	0.9 [0.4, 2.1]	0.82
SpO_2_ < 90% in ambient air, *n* (%)	93 (62%)	91 (61%)	1.0 [0.8, 1.3]	0.81
Readmission to or prolonged stay (>36 h) in the intensive care unit/high-dependency unit for respiratory problems, *n* (%)	27 (18%)	27 (18%)	1.0 [0.9, 1.1]	1.00

Values are expressed as *n* (%) or medians [interquartile range]. The test methods included the *χ*^2^ test, Fisher test and Mann–Whitney U test. *p* < 0.05 was considered statistically significant. *Denotes statistically significant (*p < 0.05) differences between two groups. MGS-2, the Melbourne group scale version 2; PPCs, postoperative pulmonary complications; SpO_2_, pulse oxygen saturation; RR, relative risk.

**Table 3:** Comparison of chest drainage.

		Aggressive intraoperative warming (*n* = 150)	Routine thermal management (*n* = 150)	RR [95% CI]	HR [95%CI]	Median difference [95% CI]	*p*-value
Chest drainage for more than 5 days, n (%)		30 (20%)	51 (34%)	0.6 [0.4, 0.9]			0.03*
Time to chest tube removal, days		4 [3, 5]	5 [4, 7]		1.4 [1.1, 1.7]		0.001*
Chest drainage, mL	POD 1	125 [40, 225]	200 [100, 300]			−60 [−100, −35]	<0.001*
	POD 2	120 [45, 210]	158 [74, 250]			−30 [−55, −2]	0.01*
	POD 3	60 [20, 120]	100 [24, 183]			−20 [−50, −5]	0.01*
	POD 5	0 [0, 30]	10 [0, 103]			0 [−5, 0]	0.001*

Values are expressed as *n* (%), or medians [interquartile range]. Test methods included *χ*^2^, Fisher, Mann–Whitney U, COX regression, and repeated-measures analysis of variance with post-hoc Bonferroni correction. *p* < 0.05 was considered statistically significant. Comparison between different time points: *F* = 136.6, *p* < 0.001, ηp^2^ < 0.001; Time*Group: *F* = 21526.2, *P* = 2.2, ηp^2^ = 0.10; Comparison between different groups: *F* = 15.6, *p* < 0.001, ηp^2^ = 0.05. *Denotes statistically significant (*p < 0.05) differences between two groups. POD, postoperative day; RR, relative risk; HR, hazard ratio.

**Table 4:** Comparison of platelet levels.

		Aggressive intraoperative warming (*n* = 150)	Routine thermal management (*n* = 150)	Mean difference [95% CI]	*p*-value
Platelet Level, x10^^9^/L	Preoperative	201 ± 57	204 ± 64	−3 [−21, 7]	0.65
	Postoperative Day 1	186 ± 59	184 ± 59	2 [−15, 12]	0.83
	Postoperative Day 3	150 ± 51	149 ± 55	1 [−13, 10]	0.83
	Postoperative Day 5	171 ± 58	178 ± 115	−7 [−31, 12]	0.54

Values are expressed as means ± SDs. Test methods used repeated-measures analysis of variance with post-hoc Bonferroni correction. *p* < 0.05 was considered statistically significant. Comparison between different time points: *F* = 111.0, *p* < 0.001; Time*Group: *F* = 0.55, *p* = 0.648; Comparison between different groups: *F* = 0.77, *p* = 0.782.

### 3.3. Other complications

There were no significant differences in the incidences of other postoperative complications between two groups. 22% patients had radiographic evidence of subdiaphragmatic free gas (33/150) in patients with routine thermal management versus 19% in those assigned to aggressive warming (29/150). The incidence of subcutaneous emphysema was 23% (34/150) with routine management group and 28% (42/150) in the aggressive management group. Chylothorax occurred in 4 patients in the routine management group and 2 patients in the aggressive management group. Intestinal obstruction occurred in 1 patient and cerebral infarction occurred in 2 patients in the routine body temperature management group. Laryngeal edema and heart failure occurred in 1 patient in the aggressive body temperature management group (Table 5).

**Table 5:** Comparison of other complications.

	Aggressive intraoperative warming (*n* = 150)	Routine thermal management (*n* = 150)	*p*-value
Extrapulmonary complications			
Chylothorax, *n* (%)	2 (1%)	4 (3%)	0.68
Laryngeal edema, *n* (%)	1 (1%)	0 (0%)	0.49
Heart failure, *n* (%)	1 (1%)	0 (0%)	0.49
Intestinal obstruction, *n* (%)	0 (0%)	1 (1%)	>0.99
Cerebral infarction, *n* (%)	0 (0%)	2 (1%)	0.50
Subdiaphragmatic free gas, *n* (%)	29 (19%)	33 (22%)	0.57
Subcutaneous emphysema, *n* (%)	42 (28%)	34 (23%)	0.29
Duration of stay in ICU, days	1 [1, 1]	1 [1, 1]	0.93
Length of stay after surgery, days	9 [8, 12]	8 [7, 11]	0.34

Values are expressed as *n* (%), or medians [interquartile range]. Test methods included *χ*^2^, Fisher, Mann–Whitney U, and repeated-measures analysis of variance. *p* < 0.05 was considered statistically significant.

In patients assigned to aggressive warming, the median postoperative ICU stay was 1 [interquartile range: 1, 1] day, and the postoperative duration of hospitalization was 9 (8, 12) days. The corresponding durations in the routine thermal management group were 1 (1) day and 8 (7, 11) days (Table 5).

## 4. Discussion

The main finding of the current study revealed that aggressive warming did not reduce PPC incidence but shortened chest drainage duration in elderly patients receiving esophagectomy. In our study of 300 patients, PPCs occurred in 18% of the aggressive warming group compared to 21% of the routine thermal management group, without reaching statistically significant. Nonetheless, aggressive warming reduced the chest drainage duration from the median of 5 days to 4 days and the number of patients having chest drainage for more than 5 days decreased from 34 to 20%, which indicated a 40% reduction. No significant differences were found in other postoperative complications.

Our study was a pre-defined sub-study of the 5,056-patient PROTECT multicenter trial (15). All patients thus qualified for the underlying trial and thermal management was per PROTECT randomization. Our sub-analysis was restricted to patients at the Shanghai Chest Hospital who were scheduled for esophagectomy, and thus likely to develop pulmonary complications. Furthermore, our sub-study outcomes differed in that we considered postoperative chest drainage, blood lactate, and extrapulmonary complications including intestinal obstruction, chylothorax, and subdiaphragmatic free gas–none of which was considered in the underlying trial.

Chronic respiratory disorders, lung reserve reduction, small airway collapse, and other anatomical or physiological changes all promote pulmonary complications. We included patients at high risk of PPCs, as indicated by minimum ARISCAT (Assess Respiratory Risk in Surgical Patients in Catalonia) scores of at least 50 points, with scores exceeding 44 points indicating high risk of PPCs (19). Furthermore, our patients were all at least 51 years old, scheduled for thoracic surgery, and had an expected operating time exceeding 3 h. It is therefore unsurprising that the overall incidence of pulmonary complications was 19%, which is consistent with previous reports of 26–48% (4–7).

Hypothermia following esophagectomy is common, and may promote infection, gastrointestinal ischemia, and lung injury–each of which increase the risk of postoperative pulmonary complications (20). Nonetheless, aggressive warming had no significant impact on pulmonary complications compared with routine core-temperature management, suggesting that core temperatures above 35.5°C are sufficient with respect to pulmonary complications which was our primary outcome. Our results are consistent with those of the underlying PROTECT trial which also found that the incidence of cardiovascular complications, surgical sites infections, and transfusions were similar at 35.5°C and 37°C.

Hypothermia directly impairs platelet (21) and immune functions, including T cell-mediated antibody production and nonspecific oxidative bacterial killing by neutrophils (22). As core body temperature decreases from 41°C to 26°C, the anti-bacterial function of neutrophils gradually decreases (23). Additionally, hypothermia reduces release of proinflammatory cytokines (TNF-α, IL-1β, IL-6) (24). Unsurprisingly, therapeutic hypothermia and consequent immunosuppression increases the risk of pulmonary infection in both trauma and postcardiac arrest patients (25, 26). As might thus be expected, a meta-analysis reports that patients randomized to therapeutic hypothermia after cardiac arrest were more likely to develop pneumonia: RR 1.15 [95% CI: 1.02, 1.30] (27). Pulmonary infections were also more common in patients with traumatic brain injury who were randomized to hypothermia: 66.7% vs. 36.7%; *p* = 0.038 (28).

In this study, the incidence of postoperative purulent sputum was 93 out of 150 (62%) in the routine thermal management group and 74 out of 150 (49%) in the aggressive intraoperative warming group (*p* = 0.03). However, it is important to note that purulent sputum is only one of the evaluation indexes for postoperative pulmonary complications. There were no significant differences between the two groups in terms of fever, white cell count, chest X-ray findings, sputum microbiology, oxygenation saturations, and ICU readmission. The MGS-2 score, which is used to diagnose PPCs, was also similar between the two groups. Based on the current results, we cannot yet conclude that aggressive intraoperative warming is beneficial in reducing the occurrence of PPCs.

Aggressive warming reduced the time of chest tube drainage from 5 days to 4 days and reduced postoperative chest drainage volume. Presumably consequently, the incidence of delayed chest tube drainage was 51/150 patients in the routine body temperature management group and 30/150 in the aggressive group, with a RR of 0.6 [95% CI: 0.4, 0.9]. We hypothesize that the observed difference in postoperative pleural effusion may be attributed to a combination of factors, including bleeding due to variations in platelet function and difference in postoperative pneumonia rates.

Coagulation profiles were not monitored, and there was no temperature-dependent difference in blood loss or transfusion requirement in the underlying PROTECT trial (15). Despite this, we conducted a retrospective analysis of postoperative platelet levels for the 300 enrolled patients based on their medical history data. Unfortunately, we did not perform routine postoperative coagulation function monitoring for all patients undergoing esophageal cancer surgery, so the retrospective analysis could not obtain the patients’ clotting profiles. The postoperative platelet levels in both groups initially decreased and then increased over time, with no significant difference between them. Consequently, we speculate that intraoperative mild hypothermia does not significantly impact postoperative platelet levels in patients. However, platelet counts only reflect changes in platelet numbers. There is considerable evidence that hypothermia decreases platelet aggregation (29) and impairs enzymes of the coagulation cascade (30), thus increasing blood loss. Improved coagulation with aggressive warming may therefore explain the observed reduction in chest drainage. Additionally, intraoperative hypothermia may aggravate lung injury during one-lung ventilation and the complex inflammatory response. Increased permeability of lung tissue vascular intima and pleura due to hypothermia may also contribute to the observed difference in postoperative pleural effusion.

The limitation of our trial is that we enrolled only 300 patients, since small trials routinely over-estimate treatment effects. In the sample size calculation, we estimated a reduction in PPCs with an aggressive rewarming strategy of 10%, but observed a final difference in incidence of 5%. While it is possible that the study was underpowered to detect a statistically significant difference, we believe that our findings are still clinically relevant. Unfortunately, the PROTECT trial has completed enrollment of all patients, and it is not possible to increase the sample size.

Our trial was not double-blinded because the perioperative teams needed to implement the designated thermal management plan for each patient. Patients also knew that they were pre-warmed. However, postoperative pulmonary complications and chest tube drainage are objective outcomes that are unlikely to be influenced by the patient’s perception of warming or by the absence of blinding to allocation. An additional limitation is that all patients were Chinese and all received general anesthesia. Results might differ in other populations, including morbidly obese patients and those having emergency surgery.

## 5. Conclusion

In summary, aggressive temperature management does not reduce the incidence of PPCs in elderly patients after curative resection of esophageal cancer; however, it may somewhat reduce the incidence and duration of prolonged chest drainage.

## Data availability statement

The raw data supporting the conclusions of this article will be made available by the authors, without undue reservation.

## Ethics statement

The studies involving human participants were reviewed and approved by the Ethics Committee of Shanghai Chest Hospital (Institutional Review Board #KS1905). The patients/participants provided their written informed consent to participate in this study.

## Author contributions

JW, QJ, YQ, and DS contributed to conception and design of the study and wrote sections of the manuscript. XL and WT organized the database. XL performed the statistical analysis and wrote the first draft of the manuscript. All authors contributed to manuscript revision, read, and approved the submitted version.

## Funding

This work was supported by the Fund of Shanghai Shen Kang Hospital Development Center Project (SHDC2020CR4063).

## Conflict of interest

DS occasionally consults for temperature-related companies, and donates all fees to charity.

The remaining authors declare that the research was conducted in the absence of any commercial or financial relationships that could be construed as a potential conflict of interest.

## Publisher’s note

All claims expressed in this article are solely those of the authors and do not necessarily represent those of their affiliated organizations, or those of the publisher, the editors and the reviewers. Any product that may be evaluated in this article, or claim that may be made by its manufacturer, is not guaranteed or endorsed by the publisher.
